# Neural implementation of musical expertise and cognitive transfers: could they be promising in the framework of normal cognitive aging?

**DOI:** 10.3389/fnhum.2013.00693

**Published:** 2013-10-22

**Authors:** Baptiste Fauvel, Mathilde Groussard, Francis Eustache, Béatrice Desgranges, Hervé Platel

**Affiliations:** ^1^INSERM, U1077Caen, France; ^2^Université de Caen Basse-Normandie, UMR-S1077Caen, France; ^3^Ecole Pratique des Hautes Etudes, UMR-S1077Caen, France; ^4^CHU de Caen, U1077Caen, France

**Keywords:** musical training, expertise, brain plasticity, cognitive transfer, aging, brain reserve

## Abstract

Brain plasticity allows the central nervous system of a given organism to cope with environmental demands. Therefore, the quality of mental processes relies partly on the interaction between the brain’s physiological maturation and individual daily experiences. In this review, we focus on the neural implementation of musical expertise at both an anatomical and a functional level. We then discuss how this neural implementation can explain transfers from musical learning to a broad range of non-musical cognitive functions, including language, especially during child development. Finally, given that brain plasticity is still present in aging, we gather arguments to propose that musical practice could be a good environmental enrichment to promote cerebral and cognitive reserves, thereby reducing the deleterious effect of aging on cognitive functions.

## INTRODUCTION

To adapt to changing environmental requirements, the brain has structural and functional dynamic properties that allow regularities to be encoded and skills to be learned and refined through regular practice (Draganski and May, 2008). In return, the efficiency of the cognitive system is partly influenced by environmental features and individual daily experiences (Plomin et al., 1999).

Mastering a musical instrument is a particularly complex multimodal and multiprocesses behavior because it requires a theoretical learning of the solfeggios rules, as well as a repeated training for translating visual codes (the written scores) into precise motor sequences and auditory events (Wan and Schlaug, 2010). Musical training and musicians have therefore been often used in studies focusing on practice-related brain plasticity. Interestingly, these investigations have brought some empirical evidences arguing that the regular commitment in musical activities does not only lead to behavioral improvements in perceptivo-motor skills, but also in a wide range of non-specific cognitive functions (Schellenberg, 2006).

In this article, we review (1) the structural plasticity effects induced by musical practice, to point out that they can be located in general cognitive related-areas outside the auditory-motor domain; (2) the functional differences that exist between musically naïve and musically trained subject when they perform auditory-motor tasks, to show that trained subjects can rely on general cognitive processes when the task become harder; (3) the transfers effect that occur from musical practice toward other cognitive functions, because it justify the use of music, notably in rehabilitation program for developmental disorders or brain injuries. Finally, (4) we present works and arguments to suggest that musical practice could also be encourage as a particularly good environmental enrichment to promote a successful brain aging.

## NEURAL IMPLEMENTATION OF MUSICAL EXPERTISE

### STRUCTURAL PLASTICITY INDUCED BY MUSICAL PRACTICE

Structural brain plasticity phenomenon (i.e., changes in cells anatomy and connections) can be longitudinally and cross-sectionally recorded in human using morphometric method of MRI data analysis (Draganski and May, 2008). In a longitudinal MRI study conducted byHyde et al. (2009), 31 children aged around 6 years were scanned before being assigned to 15 months of either individual keyboard lessons or a non-instrumental music class. Whereas no structural differences were observed before the experiment, those children who had learned to play the keyboard exhibited significantly greater increases in gray matter volume after the lessons within the primary motor cortex (right pre-central gyrus) and motor-related areas (part of the corpus callosum), as well as within the primary auditory cortex (right Heschl’s gyrus). The extent of the rearrangements in these motor and auditory areas was correlated with improvements in finger tapping and auditory frequency discrimination tasks, respectively. Interestingly, the authors also found a greater increase in gray matter volume in brain regions dedicated to the integration of multimodal information (bilateral frontal areas and left pericingulate gyrus). In the context of musical practice, these regions may sustain the translation of the visual score into motor sequences and auditory-induced emotional events (Hyde et al., 2009).

Cross-sectional studies conducted with adult expert musicians have confirmed this anatomical brain shaping on several occasions. Adult violinists have a more highly elaborated topographic representation of their left hand in the right somatosensory cortex (Elbert et al., 1995). Moreover, the gray matter volume of the primary motor cortex (pre-central gyri) has been shown to increase in relation with the degree of musical expertise (Gaser and Schlaug, 2003), with a left-hemisphere advantage for pianists and a right-hemisphere one for string players (Bangert and Schlaug, 2006), illustrating the close link between anatomical changes and training demands. Regarding the auditory cortex,Bermudez and Zatorre (2005) found greater gray matter volume in the right superior temporal gyrus of 43 adult musicians compared with non-musicians. Beyond the motor and auditory cortices, other local gray matter increases, possibly induced by score reading, have been found in the visuospatial and visuomotor areas (right fusiform gyrus, left intraparietal sulcus;James et al., 2013) and left inferior temporal gyrus (Gaser and Schlaug, 2003), as well as in multimodal association areas (right superior parietal gyrus;Gaser and Schlaug, 2003), and general cognitive-related areas (left inferior frontal gyrus;James et al., 2013).

Structural brain plasticity phenomenon also appears to take place in white matter tracts. The corpus callosum exhibits enlargement in musicians because they engage more frequently in bimanual behavior (öztürk et al., 2002;Hyde et al., 2009), and possibly because this structure is involved in visuoauditory processes too (Bengtsson et al., 2005). Bengtsson and colleagues also found an increase in the diffusivity value of the internal capsule of the corticospinal tract, and a positive correlation between hours of musical practice and fiber tract diffusivity in frontal areas and the right arcuate fasciculus, which are crucial for language processing, and have been shown to be larger and more structured in musicians, especially singers (Halwani et al., 2011).

To resume, performing music involves elaborated, coordinated, and rules-based motor, auditory, and visual skills. Regular musical training therefore leads to an anatomical shaping of auditory, motor and visual-related areas, but some results showed that brain regions involved in more general non-musical cognitive processes, such as language, attention, or executive functions, are also affected by musical training-related structural plasticity (Hyde et al., 2009;James et al., 2013). This indirectly argues that these later functions could also play a crucial role in musical expertise achievement.

### FUNCTIONAL PLASTICITY INDUCED BY MUSICAL PRACTICE

The impressive auditory skills and manual dexterity of musicians are not solely explained by the *anatomical* remodeling of auditory and motor-related areas, as they also exhibit *functional* brain differences with non-musicians when they perform tasks similar to music exercises (e.g., finger tapping tasks, tonal, or temporal auditory discrimination tasks).

fMRI studies have shown that when professional pianists and violinists are asked to perform complex uni- or bi-manual tapping tasks, they exhibit reduced activation of the primary and secondary motor areas (Jäncke et al., 2000;Lotze et al., 2003) as well as the supplementary and pre-motor areas (Krings et al., 2000), compared with matched controls. The authors of these studies concluded that motor skills are more automatized, and thus less costly for musicians, who need therefore to recruit fewer neurons for performing as well as controls subjects do. This automatization frees up resources that allow for better spontaneity and flexibility, as reflected in the greater activation of bilateral pre-frontal and parietal areas (Lotze et al., 2003;Landau and D’Esposito, 2006).

Regarding auditory skills, once again, besides the structural shaping of the auditory areas, particularities in neural responses have been recorded when expert musicians process musically relevant auditory stimuli. They have an enhanced cortical representation of the musical scale tones in the tonotopic map of the auditory cortex (Pantev et al., 1998) that leads them to exhibit greater expectancy and more effective attentional processes for musical sounds. Indeed, when they have to identify a sound with a deviant frequency (pitch) or rhythm embedded in a sequence of standard sounds, they display shorter latencies and/or higher amplitudes of the electrophysiological components known to reflect conscious sound discrimination and target detection [N2b and P3;Tervaniemi et al., 2005; Late Positive Component (LPC);Besson et al., 1994; P3,Ungan et al., 2013].

Musicians’ enhanced cortical representations of musical rules turn also in a pre-attentive memory-based processing for musical sound encoding, maybe through the auditory corticofugal pathway. Indeed, modulations in both cortical and subcortical electrophysiological responses to the perception of musical irregularities have even been recorded when musicians’ attention is diverted away from the stimuli. It is reflected in the appearance or increase of the MisMatch Negativity (MMN) component in the auditory cortex of musicians versus non-musicians when they are exposed to a melodic contour or interval change (Koelsch et al., 1999;Fujioka et al., 2004), and in the modulation of the Early Right Anterior Negative (ERAN) component when the changes consist of more complex musical irregularities (e.g., unexpected chords;Koelsch et al., 2002).

Concerning the subcortical level, musicians display faster neural synchronization and stronger brainstem encoding of chord arpeggios in both in tune and out of tune conditions. Interestingly, the magnitude of the brainstem response is predictive of the participants’ performances in a pitch discrimination task (Bidelman et al., 2011).

Neuroimaging studies using fMRI have also been designed to explore whether musically trained individuals process the auditory aspects of music differently from non-musicians. When they are passively exposed to music or have to judge whether chords are consonant or dissonant, musically trained participants display a right to left shift in the activation of auditory temporal areas (Ohnishi et al., 2001) and frontal and inferior parietal areas (Minati et al., 2009). According to the authors, this suggests the use of more abstract and analytical strategies by the experts. Studies featuring pitch comparison tasks that involve greater working memory load (Gaab and Schlaug, 2003) or cognitive control and updating in working memory (Pallesen et al., 2010) report that, compared with non-musicians, musically trained participants recruit fewer early perceptual areas, and more auditory working memory-related areas (right posterior temporal and supramarginal gyri and bilateral superior parietal areas) or associative areas (right pre-frontal, parietal lateral, anterior cingulated, and dorsolateral frontal cortices). The more difficult the task is, the more the experienced musicians resort to these areas and the more their performances are better compared with those of controls (Oechslin et al., 2012). Finally, regarding musical semantic memory, a study conducted in our laboratory revealed that when musicians have to rate their familiarity with a melody, several autobiographical episodic memory-related areas are activated more strongly compared with non-musicians (bilateral hippocampus, visual primary cortex, cingulate cortex, and bilateral superior temporal areas;Groussard et al., 2010). This suggests that, owing to their musical experience, musicians engage self-referential processes to perform the task and gain access to richer and more vivid sensory details.

Moreover, musical expertise also leads to an auditory-motor coupling, as reflected by the activity displayed within the primary motor cortex when musicians hear a piece they have learned to play (Haueisen and Knösche, 2001;Bangert et al., 2006). Conversely, when they tinkle on a mute keyboard, the temporal areas dedicated to hearing are activated. In the same way, musical expertise is also built on auditory-visual and visuomotor associations. Indeed, it has been shown that when an atonal event (a wrong note) is written on a score, musicians are able to anticipate it, as reflected in their behavioral performance and physiological response to this event (Schön and Besson, 2005). Finally, when motionless pianists imagine themselves playing a piece from a score, they display the same pattern of neural activity within the secondary and associative motor areas as they do during an actual instrumental performance, although the degree of activation is reduced and does not concern the primary motor cortex (Meister et al., 2004).

To sum up so far, the expert auditory and motor performances of musicians are sustained by the functional reorganization of typical underlying neural processes, due to automation and better memory-based (top-down) processing. This makes the basic auditory and motor skills less costly for musicians, thereby allowing them to allocate resources to strategic processes when tasks become harder. Similar effects have also been observed for expert performance in other activities such as object and pattern recognition by chess master players (Bilalic et al., 2012) or working memory-related tasks (Guida et al., 2012).

## COGNITIVE TRANSFER OF MUSICAL TRAINING

Musical hearing and practice seem not to be sustained by specific brain areas, but involve rather general pre-existing skills and cognitive functions. As musical learning put a high demand on it, it can result in transfers of improvements from one activity (music making) to others (e.g., language skills, executive functions).

### LANGUAGE SKILLS

There is a longstanding debate about the division or sharing of the brain substrates of language and music. Although some famous neuropsychological cases of double dissociation have been reported (Stewart et al., 2006), we know that music and language perception and production share a lot of features, ranging from the basic sensorimotor level to auditory-cognitive processes. Indeed, it appears that humans perceive these two auditory stimuli using the same acoustic cues (rhythm, pitch, and timbre) and relying on similar resources for syntactic integration processing or memory. Furthermore, both language and music have a visual form of notation that allows them to be red or written, and producing either of these two behaviors involves auditory-motor coupling. According to Patel’s OPERA hypothesis, transfers from musical training to language skills occur because (i) there is an anatomical overlap in their brain networks, (ii) musical practice implies more precision in processing features shared with language, (iii) this practice takes place in a repeated manner, and is associated with (iv) more focused attention, and (v) emotion (Patel, 2011).

Although they are less central to language than to music, pitch modulations are still important for speech in order to convey emotion. Cross-sectional (Magne et al., 2006), and longitudinal studies (Moreno and Besson, 2005) found that musically trained children were better at detecting pitch violations in a foreign or native language than their musically untrained counterparts. Moreover, the authors reported a cortical Late Positive Component (LPC) in response to pitch violations that was far less pronounced, or even absent, in the untrained children. Rather similar results have been reported for adults, with musicians displaying a shorter latency of this LPC when exposed to prosodic incongruities in foreign sentences (Schön et al., 2004;Marques et al., 2007).

Musicians also process the temporal features of speech sounds differently than non-musicians. Faster cortical neural responses to voice onset time, as well as to vowel or syllable duration, have been attested both attentively and pre-attentively for children who take part in music lessons (Chobert et al., 2012). As electrophysiological modulations have also been found at the brainstem level (Musacchia et al., 2007;Wong et al., 2007),Kraus and Chandrasekaran (2010) suggest that transfer effects from processing acoustic cues in a musical context to processing them in a speech context are mostly due to the reinforcement of the auditory corticofugal pathways (as in the reverse theory hypothesis that states that long-term cortically stored representations guide early perceptual encoding via descending pathways and top-down processes). The overtraining of musicians to detect, sequence, and encode relevant aspects of musical sound patterns shared with speech endows them with heightened phonological awareness.

Apart from the benefit of perceiving prosody in native and foreign languages, phonological awareness is also crucial for learning tone languages (Milovanov and Tervaniemi, 2011), hearing speech in noise, and reading skills (Strait and Kraus, 2011). This make music lessons potentially well suited to the rehabilitation of children with reading impairments such as dyslexia (Strait and Kraus, 2011).

Another point that music and language have in common is that they consist of auditory elements that unfold over time according to complex rules referred to collectively as syntax. Although the neural representations of these regularities are stored in different parts of the brain, their processing requires the same limited neural resources, allowing for transfer effects (Patel, 2011). When exposed to syntactic incongruities in speech, musicians have different Event-Related Potential (ERP) responses compared with non-musicians. These responses are more bilateral in adult musicians (Fitzroy and Sanders, 2013), and appear earlier during the development in children who participate to music lessons (Jentschke and Koelsch, 2009).

We have seen that regular musical training results in the reinforcement of auditory-motor coupling. This coupling is also essential for speech, and its stimulation through musical practice seems to contribute to the rehabilitation of stroke patients with language impairments (Rodriguez-Fornelis et al., 2012).

Finally, it seems that the short-term storage and manipulation of speech relies partly on the same neural networks and cognitive mechanisms in working memory as music (Besson et al., 2011;Schulze et al., 2011;Strait and Kraus, 2011). Regarding long-term episodic storage, a study conducted in our laboratory showed that the brain areas engaged in musical episodic retrieval match those known to be activated for the retrieval of non-musical stimuli (bilateral middle and superior frontal gyri and pre-cuneus;Platel, 2005). This could explain why musicians, who frequently use short- and long-term memory resources for processing music, often perform better than non-musicians on verbal working memory tasks (Bialystok and DePape, 2009), and sometimes on verbal episodic memory tasks, too (Chan et al., 1998).

To conclude, it appears that the brain areas thought to be dedicated to language and speech processing are rather involved whenever a behavior calls for fine-grained auditory analysis and implies an auditory motor coupling. Because musical experts rely on these brain mechanisms to process and play music, it turns to implications of language-related areas for processing music and to transfers from musical practice to language processes.

### EXECUTIVE FUNCTIONS AND IQ

The positive effects of musical practice on cognition are not restricted to auditory and language skills. Considering literature, it seems that playing or learning music is linked to better performances on an astonishing range of cognitive measures. Even after taking the effects of potential confounding variables (e.g., family income, parents’ education, and involvement in non-musical activities) into account,Schellenberg (2006) observed a positive association between the duration of music lessons in childhood and full-scale IQ results, as well as academic achievement. No evidence was highlighted for a particular strong correlation between musical training and one specific IQ subtests. Moreover, all the correlations disappeared when the IQ score was held constant, arguing for a homogenous and non-specific effect of musical training on intelligence. To certify a little the causal link, the authors replicated their findings in a follow-up study where children were assigned either to music, drama, or no lessons groups (Schellenberg, 2004). After the lessons, the improvement in IQ performances was significantly greater in the groups that had taken music lessons than in the other groups. Given thatSchellenberg (2006) subsequently failed to find any effect on IQ test results when full-time music students were compared with non-musicians studying psychology, law, or physics, he concluded that musical learning is an extra-scholar, but scholar-like activity that requires more concentration, attention, and discipline than other everyday leisure activities. Therefore, according to this theory, music lessons could enhance the ability to plan and make decisions, correct errors, ignore irrelevant or distracting information, produce novel responses and avoid habitual ones, and cope well in difficult situations. These skills, referred to as *executive functions*, serve all the other cognitive domains and all kinds of learning. Accordingly, just like school learning does, taking music lessons as an out-of-school activity should enhance IQ in a non-specific manner (Hannon and Trainor, 2007).

To conclude, according to some authors, taking music lessons during childhood seem to act as a particularly powerful environmental enrichment to potentiate all cognitive development through the potentiation of general cognitive resources such as executive functions.

## MUSICAL PRACTICE AS A SHIELD AGAINST AGING

### CEREBRAL PLASTICITY AND COGNITIVE AGING

With the ongoing increase of life expectancy in industrialized countries, cognitive and brain aging have become key issues in neuropsychology. It is known that interindividual differences regarding cognitive performances increase with aging and that clinical consequences of aging-related cerebral atrophy differ consistently from one individual to another (Villeneuve and Belleville, 2010). This has led researchers to come up with the concepts of brain and cognitive reserve. *Brain reserve* is based on the brain’s anatomical characteristics and the fact that a higher gray matter volume counteracts atrophy and delays the appearance of the first clinical symptoms. Co*gnitive reserve* refers to neurocognitive mechanisms, such as enhancing the efficiency of the brain networks engaged to carry out a task, or using either supplementary or entirely alternative networks, reflecting recourse to compensatory strategies. These functional changes must allow maintaining efficient cognitive performances in the face of age-related physiological disturbances (Stern, 2009). Researchers have shown that the quality of such reserves is partly determined by early environmental features such as educational level, but also actual environmental variables, such as occupational level. In this framework, we think that musical activities could be a particularly suited occupation to promote brain and cognitive reserves. Indeed, (1) as we have seen, results from children and adults subjects indicate that musical practice is both a multimodal and a multiprocess activity, leading to wide cerebral plasticity phenomenon and putting heavy demands on executive functions that might help in this way to promote cognitive development (Schellenberg, 2006;Hannon and Trainor, 2007). It is not obvious that researcher will find similar results in elderly subjects (Strobach et al., 2012a,b), but there is good hopes that musical activities could be appropriate in the field of normal cognitive aging, where deterioration mostly affects executive functions (Verhaeghen, 2011), partly because of the neural disconnection that disrupts the functional integration of multiple systems (Madden et al., 2011). (2) Music (as other leisure activities) owns implicitly several characteristics of rehabilitative paradigms (e.g., gradual increment in task difficulty, motivation and arousal states elicited by the exercise, useful performance-related feedback and rewards;Green and Bavelier, 2008) that greatly increase training efficacy, generalization, and flexibility. Moreover, (3) music is not only a cognitively costly activity, but also an artistic occupation, a new field of research try now to demonstrate that musical enjoyment and chills are sustained by the release of chemical neurotransmitters (Blood and Zatorre, 2001:Salimpoor et al., 2011) and regulate some hormones level (Boso et al., 2006;Fukui and Toyoshima, 2008). Interestingly, some of these neurochemicals mechanisms could influence brain healing in positive way by partly controlling neurogenesis (Fukui and Toyoshima, 2008) and synaptogenesis (Kuo et al., 2008), as well as blood pressure level (Ramchandra et al., 2005). But the speculation that music could favor brain healing through its esthetic experience-related neuroendocrine mediators remains to be well empirically established.

At this moment, to our knowledge, only two studies have focused on the influence of musical practice on cognitive aging quality. One study, comparing professional musicians with amateur musicians and control participants aged from 60 to 83 years old, revealed that performances on the deferred recall of a geometrical shape, verbal denomination, and executive function tasks differed significantly in favor of professional musicians, with the amateur musicians’ performances lying midway between those of the professional musicians and the controls (Hanna-Pladdy and MacKay, 2011). As it has been proposed for child development, the authors argued that musical practice has a strong effect on executive functions, indirectly improving a wide range of mental processes. In the other study, individualized piano lessons were given to non-musicians aged from 60 to 85 years (Bugos et al., 2007). After 6 months of lessons, those participants who had been assigned to piano lessons exhibited significant improvements in executive tasks (Trail Making Test and WAIS codes).

To resume, among others determinants, socio-cognitive lifestyle behavior influences the quality of cognitive aging, through brain and cognitive reserve potentiation. We think that theoretical arguments (e.g., ecological nature of musical training and “chemical substrate” of musical experiences) as well as empirical results from studies conducted with children and adults subjects must encourage works aiming at validate its use in the field of aging [as done byHanna-Pladdy and MacKay (2011) andBugos et al. (2007)].

## CONCLUSION

To conclude, music is built on and constrained by the human brain and its cognitive capacities. Becoming a musician places a heavy demand on these capacities, in engaging them simultaneously and in a coordinated manner. Through regular practice, behavioral improvements are sustained by anatomical and functional brain changes, as well as by the application of strategic processes. Transfers occur from regular musical practice to non-musical skills, such as language, that rely upon the same neural resources and cognitive mechanisms. Moreover, during childhood, some authors argue that music lessons act as a particularly comprehensive source of environmental enrichment for promoting general cognitive development, perhaps through executive functions potentiation.

During aging, the main cognitive difficulties seems to deal with executive processes (Verhaeghen, 2011), therefore, there is a good reason to speculate that musical practice could also have a positive influence on cognition in this period of life. In fact, preliminary studies focusing on musical practice and cognition in elderly subjects tend to confirm this assumption (Bugos et al., 2007;Hanna-Pladdy and MacKay, 2011), but others well-controlled and comparative studies are needed to (1) confirm that musical activities engagement during life has a positive influence on cognitive efficiency during aging, (2) specify when, how, and under which conditions this influence occurs, (3) confront this influence with those of other leisure activities or cognitive intervention programs. If these investigations are conclusive, it would validate a little more the status of music as a “transformative technology of the mind” that benefits the very brain which created it (Patel, 2010).

## Conflict of Interest Statement

The authors declare that the research was conducted in the absence of any commercial or financial relationships that could be construed as a potential conflict of interest.
